# Supplementary figures and images for: DLK orchestrates a modular transcriptional response to axon injury with separate roles for Fos and Jun
Source: PLoS Genet. 2025 Dec 1;21(12):e1011969. doi: 10.1371/journal.pgen.1011969 (PMC12680358; doi:10.1371/journal.pgen.1011969)

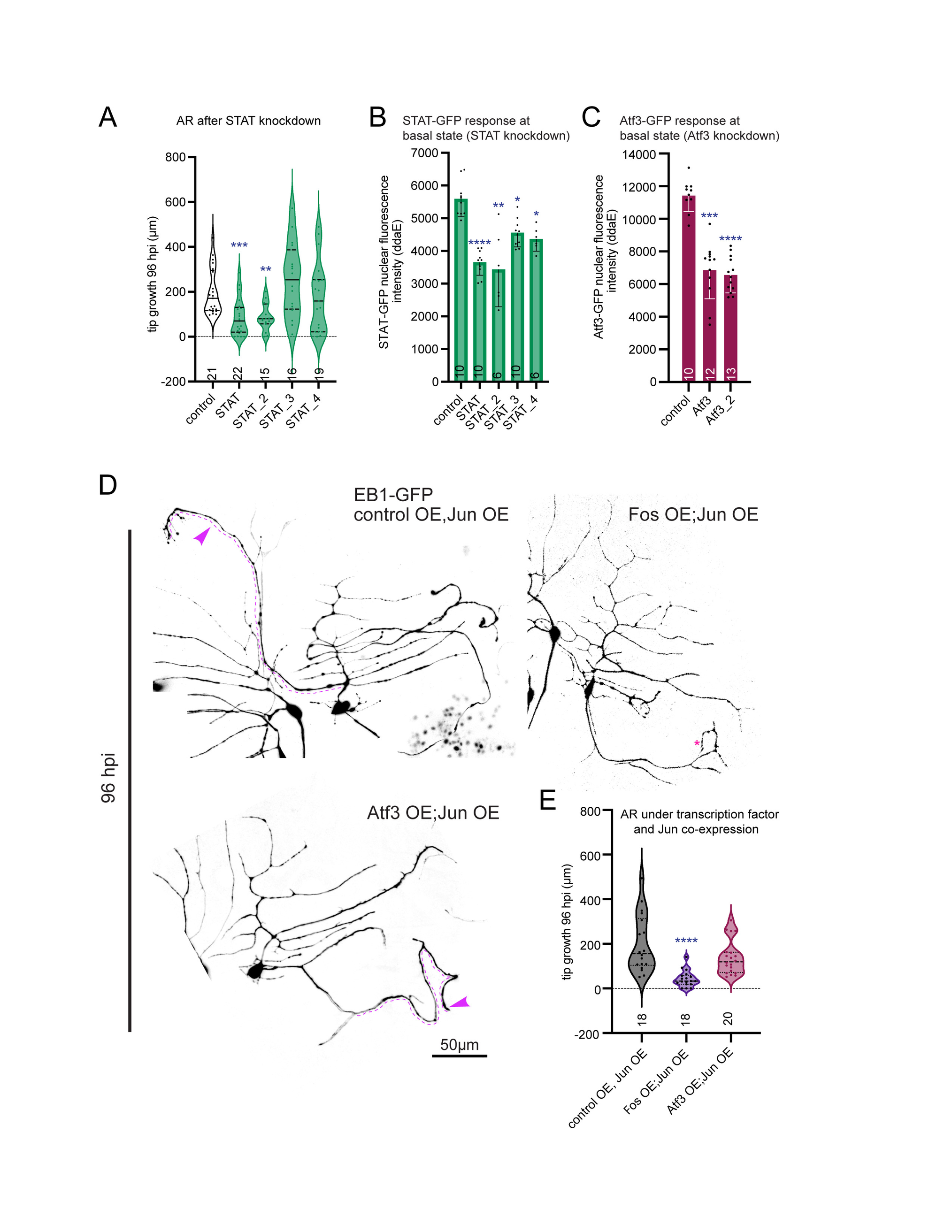

Supplement: S1 Fig — (A) Quantification of effect of multiple STAT RNAi lines on new axon tip growth at 96 hpi. The control dataset has been reused from Fig 1C. (B) Quantification of knockdown efficiency of STAT RNAi lines used in (A) tested using STAT-GFP tester line. (C) Quantification of knockdown efficiency of Atf3 RNAi lines tested using Atf3-GFP tester line. (D) Representative images of proximally axotomized class I ddaE cells at 96 hpi in different co-overexpression genetic backgrounds; all conditions include Jun overexpression. (E) Quantification of the new axon tip growth at 96 hpi, showing that co-expressing Fos and Jun significantly reduce axon regeneration compared to the control where Jun is co-expressed with control (UAS-iBlueberry). To determine statistical significance, Kruskal-Wallis one way ANOVA test was performed. The thick line represents the median, while the dashed lines represent first and third quartiles in the plot (A,E). Error bars in B and C represent SD. Numbers above the genotype are number of animals tested. *p < 0.05, **p < 0.01, ***p < 0.001, ****p < 0.0001. (TIF) [file pgen.1011969.s001.tif]

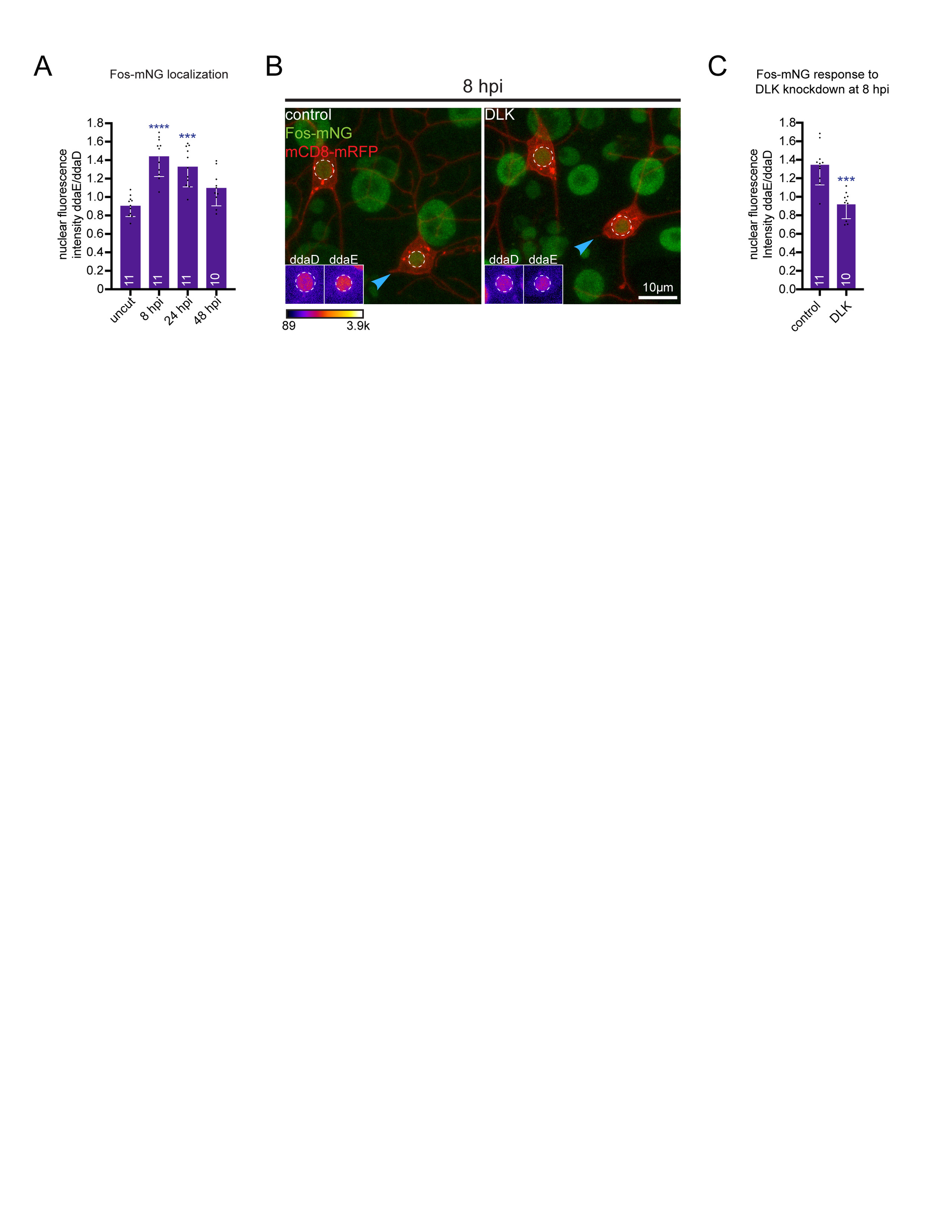

Supplement: S2 Fig — (A) Quantification of Fos-mNG nuclear fluorescence intensity in ddaE normalized to ddaD at basal state, 8 hpi, 24 hpi and 48 hpi using a second Fos-mNG fly line. (B) Representative images showing Fos-mNG at 8 hpi in control and DLK RNAi conditions. (C) Quantification of nuclear Fos-mNG fluorescence in ddaE (injured) normalized to ddaD (uninjured) at 8hpi in control and DLK RNAi. Knockdown of DLK reduces the injury-induced increase in Fos-mNG observed at 8 hpi in control. Kruskal-Wallis one way ANOVA test and Mann-Whitney test were performed in A and C respectively to determine statistical significance. The error bars represent SD. Numbers in the bars represent the number of animals tested. ***p < 0.001, ****p < 0.0001. (TIF) [file pgen.1011969.s002.tif]

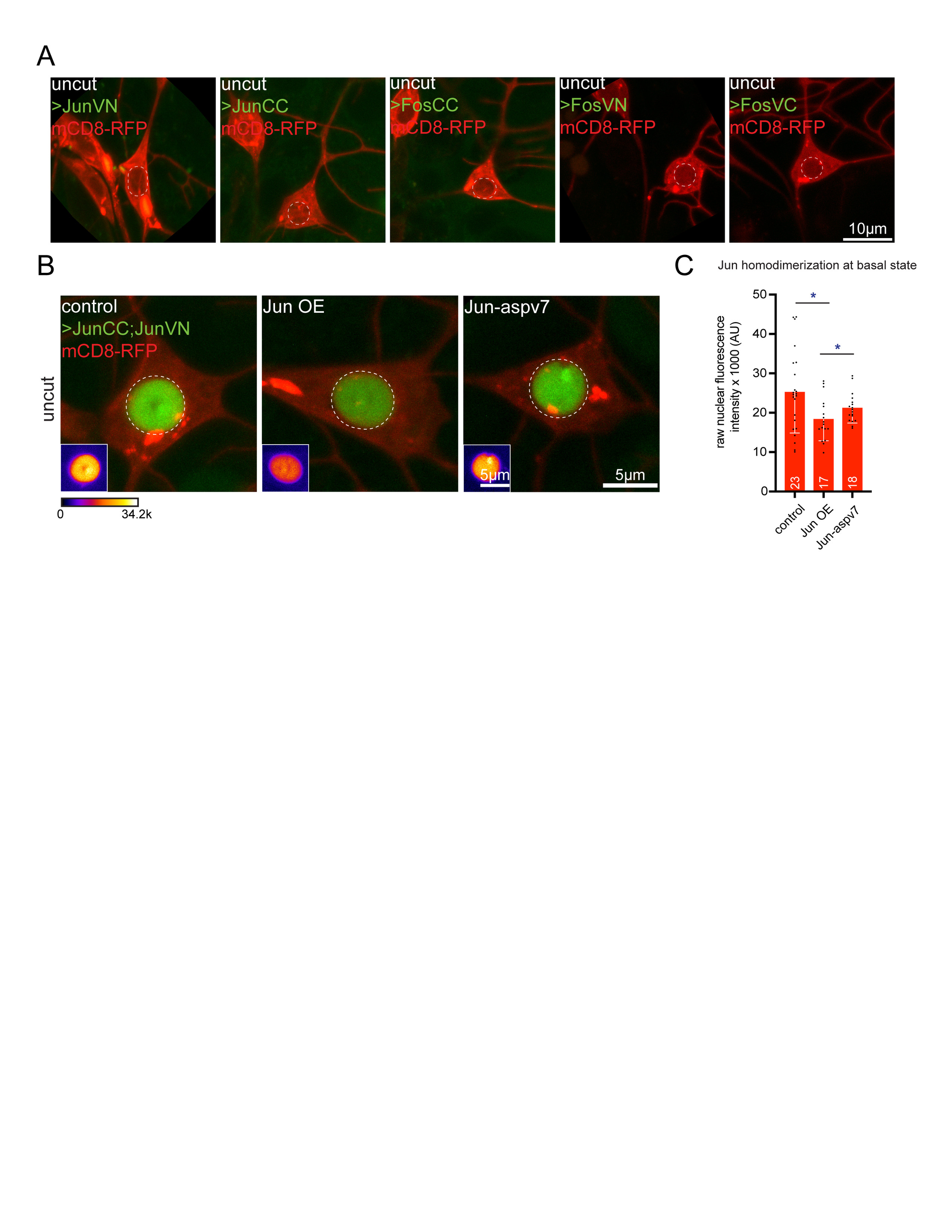

Supplement: S3 Fig — (A) Representative images of BiFC fragments attached to Jun or Fos, expressed in Class I sensory neurons showing no fluorescence at the basal state, validating that the fragments do not fluoresce without complementation. (B) Representative images of competition-based BiFC assay assessing the impact of overexpressed wild-type Jun and phosphomimetic Jun on JunVN-JunCC BiFC signal at basal state. (C) Quantification of nuclear fluorescence intensity of Jun homodimer BiFC signal at the basal state shows that phosphomimetic Jun does not compete with JunVN and JunCC as much as wild-type Jun. Insets in (B) show region of interest (indicated by dashed white circle) rendered with fire LUT for the BiFC channel. For statistical analysis, Kruskal-Wallis one way ANOVA test was used. Error bars represent SD; sample number is shown on the bar. *p < 0.05. (TIF) [file pgen.1011969.s003.tif]

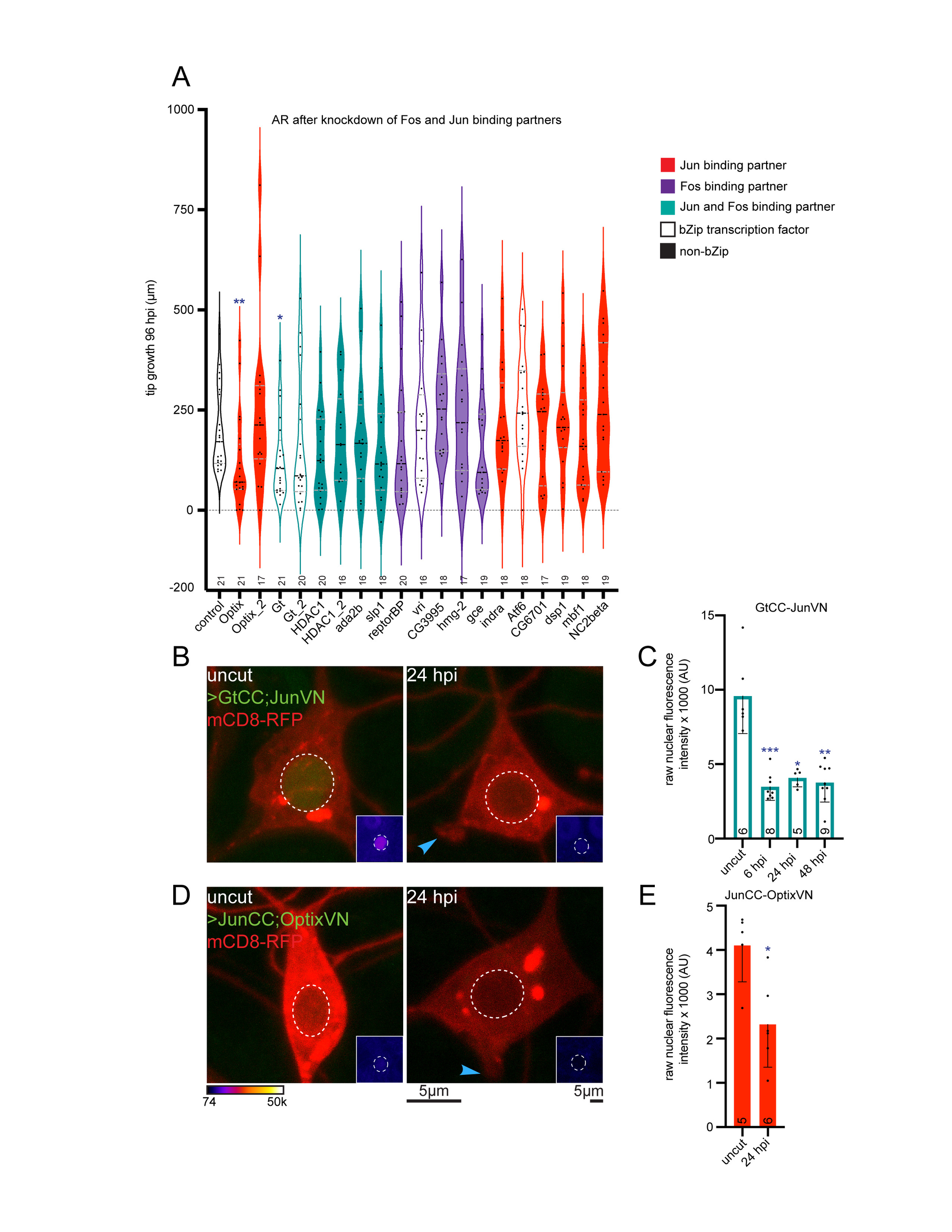

Supplement: S4 Fig — (A) Genetic screening of RNAi lines demonstrating the impact of their knock down on new axon tip growth at 96 hpi in class I ddaE neurons. Gt and Optix RNAi significantly reduced axon regeneration. The control dataset has been reused from the control in Fig 1C. (B) Representative images of GtCC-JunVN BiFC in ddaE neuron, showing reduction in heterodimerization post axon injury. (C) Quantification of nuclear fluorescence intensity of BiFC signal at the basal state and 6, 24, 48 hpi when GtCC was co-expressed with JunVN. (D) Example images of JunCC- OptixVN BiFC in Class I ddaE neurons at the basal state and 24 hpi. (E) Quantification of nuclear fluorescence intensity of BiFC signal, showing that Optix and Jun interaction reduces at 24 hpi. The blue arrows represent cut site, and the white dashed circles represent the region of interest used to quantify BiFC signal in (B) and (D). Statistical analysis in (A) was performed using Kruskal-Wallis one way ANOVA test. In the plot, the thick line represents the median, while the dashed lines represent the interquartile range (A). Error bars represent SD (C, E); sample numbers are indicated for each condition. *p < 0.05, **p < 0.01. (TIF) [file pgen.1011969.s004.tif]

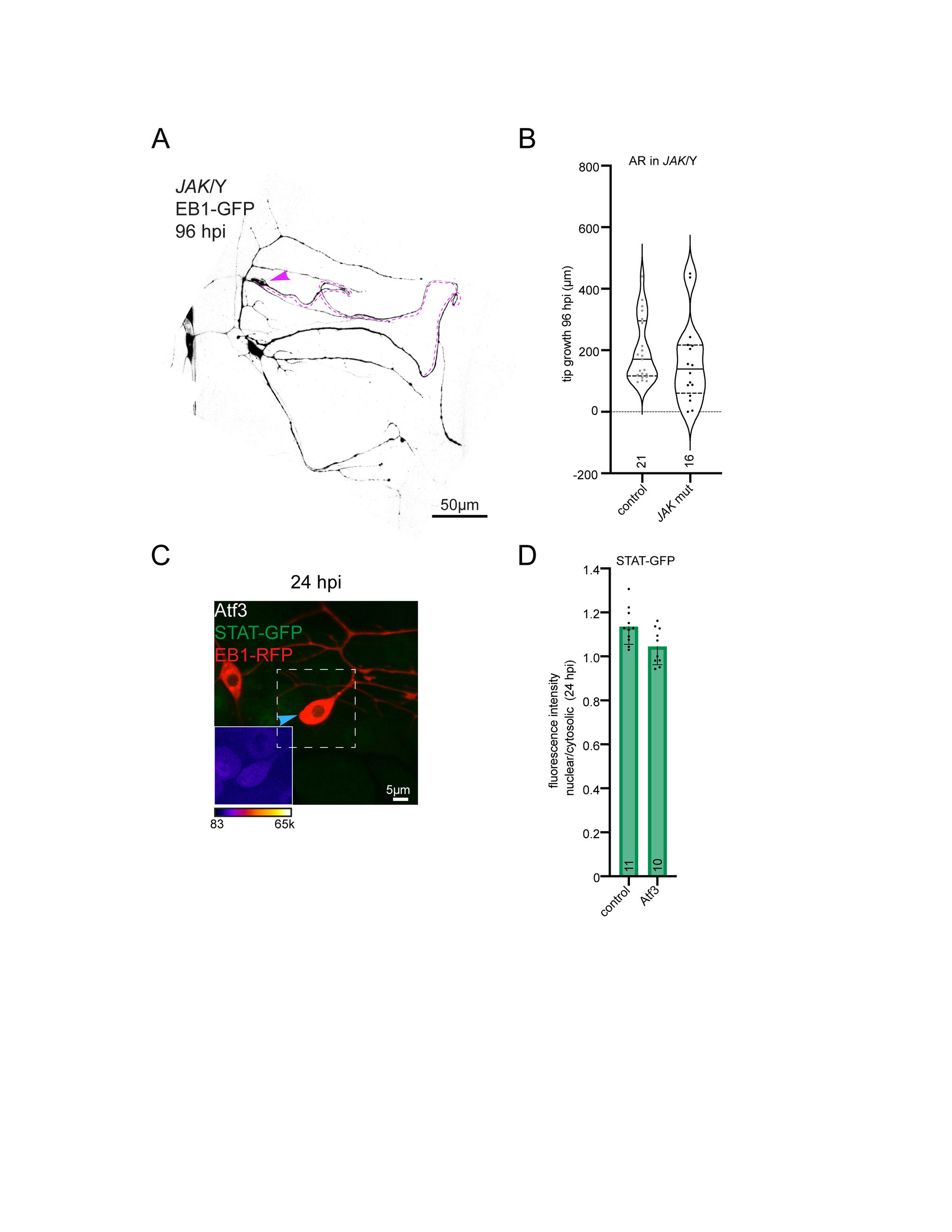

Supplement: S5 Fig — (A) Representative image of axon regeneration (AR) in class I ddaE neuron at 96 hpi in JAK mutant after proximal axotomy. (B) Quantification of axon tip growth in JAK mutant compared to control. The control dataset has been reused from Fig 1C. (C) Example image of STAT-GFP 24 hpi in class I ddaE neuron in Atf3 knockdown background. (D) Quantification of nuclear-to-cytoplasmic STAT-GFP fluorescence intensity showing that Atf3 RNAi does not have any effect on nuclear accumulation of STAT-GFP at 24 hpi. The control used for this comparison has been reused from Fig 6D. Blue arrow in (C) show axon cut site, and the inset is visual representation of the regions marked in dashed square in (C), created using fire LUT. In the plot, the thick line represents the median, while the dashed lines represent first and third quartiles (A), error bars represent SD (D), sample numbers are shown for each condition. Kruskal-Wallis one way ANOVA test. (TIF) [file pgen.1011969.s005.tif]

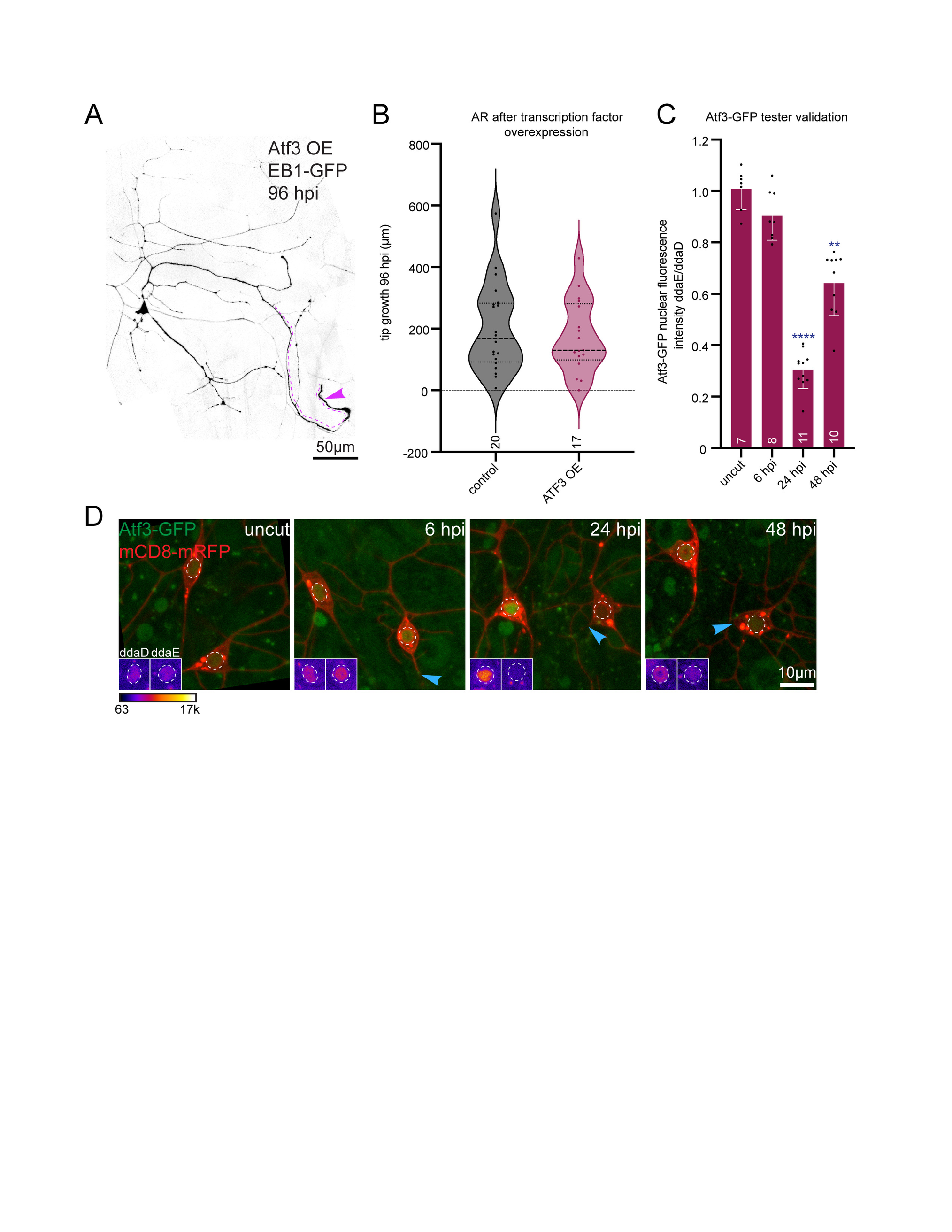

Supplement: S6 Fig — (A) Representative image of axon regeneration at 96 hpi when Atf3 is overexpressed in class I sensory neurons. (B) Quantification of new axon tip growth at 96 hpi, showing that Atf3 overexpression has no effect on axon regeneration compared to the control (UAS-iBlueberry) which has been reused from Fig 2D. (C) Atf3-GFP tester validation, showing quantification of Atf3-GFP fluorescence intensity in the nucleus of ddaE normalized to the adjacent ddaD, at basal condition and after ddaE axon was cut at 6, 24 and 48 hpi. The 24 hpi dataset includes a subset of control data in Fig 7D. (D) Representative images showing Atf3-GFP localization at basal state, 6 hpi, 24 hpi and 48 hpi using the Atf3-GFP tester. The blue arrows represent cut site, and the white dashed circles represent the region of interest. Statistical analysis in (A) was performed using Kruskal-Wallis one way ANOVA test and error bars in the graphs represent SD (A). In the plot (C), the thick line represents the median, while the dashed lines represent first and third quartiles. Sample numbers are shown for each condition. **p < 0.01, ****p < 0.0001. (TIF) [file pgen.1011969.s006.tif]
