# Supplementary material for: DLK orchestrates a modular transcriptional response to axon injury with separate roles for Fos and Jun
Source: PLoS Genet. 2025 Dec 1;21(12):e1011969. doi: 10.1371/journal.pgen.1011969 (PMC12680358; doi:10.1371/journal.pgen.1011969)
Supplement: Attachment — Submitted filename: Reviewers comments_4.docx [file pgen.1011969.s010.docx]

We thank the reviewers for their thoughtful reading of the manuscript and suggestions for supporting experiments and clarifications. In response the comments we have updated manuscript text, reorganized figures and added the following new data:

We have tested axon regeneration using additional RNAi lines for STAT, Atf3 and Slbo (Figure 1C).

We have assessed knockdown efficiency of different STAT and Atf3 RNAi lines to help interpret the axon regeneration phenotypes with these lines (Supp. Figure. 1A-C).

We have tested the effect of overexpressing Fos and Jun on Atf3-GFP nuclear fluorescence intensity (Figure 7E and F).

We tested Fos-nMG and Jun-mNG lines for function of the tagged proteins be generating homozygous viable lines (described in Results).

We have added an additional timecourse of Fos-mNG nuclear dynamics following axon injury using one of the validated viable lines (Fig. 3B and C).

We show that Fos nuclear accumulation after axon injury depends on DLK (Supp. Fig. 2B and C).

A point-by-point response to all of the comments is below. Again, we are very grateful to the reviewers and think that their input has allowed us to strengthen the manuscript.

Reviewer #1: In this manuscript entitled “DLK orchestrates a modular transcriptional response to axon injury with separate roles for Fos and Jun” by Mahata and colleagues, the transcriptional network downstream of DLK in the context of axon regeneration is explored. The authors use Drosophila larvae, in which Class I ddaE sensory neurons are axotomized to study the regenerative capacity. They employ the Gal4/UAS system to cell-autonomously manipulate various candidates to test their requirement for regeneration and the temporal sequence in which these candidates enter or exit the nucleus. Drosophila is an excellent model system for dissecting the regenerative capacity of sensory neurons, and gaining insight into the underlying mechanisms may provide novel insights for developing therapeutic approaches in humans.

In the current manuscript, the authors convey the following observations:
- Fos, Jun, STAT, Atf3, and Slbo are required for axon regeneration of sensory neurons.
- Fos controls early injury responses independently of Jun, using puc-GFP as a reporter of early responses.
- An increase of nuclear Fos following injury due to Fos homodimerization, while Fos/Jun hetero- and Jun/Jun homo-dimerizations are reduced.
- DLK/Jun activation disrupts Jun homodimerization.
- Nuclear STAT accumulation is negatively regulated by Fos, but requires Jun.
- The nuclear dynamic of Atf3 is regulated by DLK, Fox, Jun, and STAT.

This manuscript would be suitable for the general readership of PloS Genetics. But in its current form, it requires extensive polishing and reworking. I have provided several major and minor comments below, which aim to improve the quality of the manuscript to ensure it is suitable for publication. They should be adressed.

Major comments (A-F):

A) In Figure 3, the authors generated a Fos-mNG reporter. It is important to perform a proof-of-concept experiment, where the nuclear Fos localization should be dependent on DLK.

We performed the suggested experiment. The new data is shown in Figure S2 B, C and supports the pathway we proposed: DLK knock down blocks nuclear accumulation of fos 8h after axon injury.

B) Lines 178-81: Quote “Thus, the five transcription factors most closely linked to DLK signaling after axon injury across multiple systems are all required for axon regeneration in Drosophila making it a useful system to understand their relationships. We chose to pursue the four transcription factors with the strongest phenotype for additional experiments.”
It would be useful for the reader if the authors mention which one of the transcription factors has the weakest phenotype and is left out for subsequent studies.

We agree and have mentioned this in the text.

C) The authors state in the text (line 335), quote: “In summary, our data suggests that Jun phosphorylation after axon injury disrupts its homodimerization, likely allowing it to partner with another bZip transcription factor.” And they phrased the legend of Figure 5 (line 1042) as follows: “DLK/JNK signaling pathway promotes Jun homodimer dissociation through phosphorylation.”
However, the effect is rather mild, and the only experiment that was perfomed used the phosphomimetic Jun variant. Further experimental support should be provided, or the conclusion in the text and the title in the figure legend should be toned down.

We agree that the phenotype observed with the phosphomimetic Jun variant is relatively mild and suggestive rather than conclusive. We have therefore moved the data to supplementary information and modified the description to reflect its weaknesses.

D) Figure 3: Why do the authors explain the knock-in schematic at the end of Figure 3, in the display item 3E? It would make more sense to provide the concept in display Item 3A and show the data in 3B-E.

We have rearranged the panels as suggested.

E) Lines 341-90: In these paragraphs, the authors reference Figure 6. First, they reference 6A, B, then 6C, D, then 6I, J, then back to 6E, F, and finally 6G, H. Why aren’t the authors adjusting the figures according to the flow of the text? Jumping back and forth between figures while reading the text adds an unnecessary level of complexity.

We understand the concern regarding the order of figure panels that may make the text difficult to follow. From the text perspective, we structured the results to first highlight the role of upstream kinases on STAT nuclear accumulation post axotomy and then move on to the downstream players- the other transcription factors. From the figure design perspective, the four panel images in Figures 6A, C and E are visually more cohesive when presented consecutively, rather than adding current 6I and J after 6C and 6D. We felt that this arrangement provides a clearer visual flow, even though the textual references are not sequential. I hope this clarifies our rationale for the current figure layout.

F) Figure 6: In the display items C and D, the examples do not match the quantification. Please correct. In the display item K, the inhibitor of Fos should be as big as the arrows of all the mediating proteins.

We have made the genotype labels the same in panels C and D and have made the inhibitor of Fos symbol bigger to match the positive arrows.

Minor comments (1-7):

1) Figure 1C: The description of the genotypes is not clear in the figure. What is the difference between the two Jun RNAi experiments (Jun v. Jun mut; Jun RNAi)? The authors should clarify this better in the text and the figure legend.

We have updated the genotype labels in the figure to try to make this clearer and added more detail about genotypes in the legend.

2) Figure 1D: To improve the clarity for non-experts, it would be good to indicate the regenerating axon (that fails to grow out), as done for the control with the purple dashed line.
We now indicate the neurite specified as an axon based on microtubule polarity (except in the case of Fos- see figure legend), but that failed to grow, using a star.

3) Line 177: The authors mentioned AR, but did not explain what the abbreviation stands for. I assume it means axon regeneration. I suggest writing it out and putting AR in parentheses.

Sorry for this oversight; it has been added.

4) Figure 7: In the display items A and B, the 6 hpi example is left out, but included in the quantification. Please correct.

It is difficult to fit everything into the main figures, but we have added a complete image series that includes 6h to the panel to Figure S6. This Supplemental Figure shows the images and quantitation for Atf3 in the slightly different genetic background used for RNAi experiments.

5) Figure S1: The genotypes are not consistent between the examples and the quantification. Also, is the Atf3 over-expression referenced in the text? It would be better to separate the Atf3 over-expression from the Jun overexpression data, either in a different supplementary figure, or in a separate display item.

We have corrected the genotype label in the graph so that it is now consistent with the example images. The Atf3 over-expression has been referenced in the lines 359-360. We chose to show present the Atf3 overexpression together with Fos overexpression for comparison purposes.

6) Figure S2: In its current form, the figure is hard to read because the examples and quantification do not match. It would be beneficial to split the display items further and provide quantification for each example. I also suggest adding insets, as done in all other figures.

We have reorganized this data. The dendrite injury condition has been moved to the graph in Figure 4F and now we have only control images showing expression of individual halves of the BiFC transgenes in this supplemental figure (now S3). Quantitation was not included for these controls as no fluorescence above background was seen.

7) Lines 983, 117, 1142, 1153, and 1164: The authors mentioned error bars in the figure legend, but the figure does not contain error bars. Do they mean the dashed lines? If so, please explain it better.

We have addressed this and have corrected the figure legends. Previously, we had mistakenly referred to error bars instead of quartiles in the violin plots. We have now corrected it and specified that the thick line represents the median, while the dashed lines indicate first and third quartiles.

Reviewer #2: In this article, the authors aimed to dissect out the roles of different bZip transcription factors which are individually described in the context of axonal injury response. Fos, Jun, ATF etc. It was known that DLK acts upstream of these transcription factors. However, it was unclear if they act independently or as part of a connected sequence. The authors show that DLK-regulated transcription factors- Fos, Jun, STAT and Atf3 are required for axon regeneration in Drosophila model, but act during different phases. They suggest that Fos is required for early axon injury responses that stabilizes the cell. Jun is activated later by DLK, independent of Fos and it regulates STAT and Atf3. This second module is needed for later injury responses including axon regeneration. This work provides interesting insights with genetically encoded fluorescent reporters of these Transcription factors. It clearly shows FOS and Jun are working in different time windows. The manuscript has a potential to be published in PLOS Genetics. I suggests the authors to address the following concerns
[1]
1) In Figure-1, the authors showed that FOS TF is required for axon regrowth using EBP-GFP microtubule reporter. Same phenotype was noticed due to loss of jun and few other TFs. However, author proposes FOS and JUN is working in two phases in axon regeneration. In the Figure-2D, the author showed that the overexpression of FOS also reduced the axon regrowth like the loss of fos. How would the opposing genetic manipulation cause same phenotype?

Based on previous work from our lab [1], we know that fos acts upstream of global increases in microtubule dynamics post axon injury, which is a hallmark of the early neuroprotective response to axon injury. We found in this earlier study that microtubule-based neuroprotection is turned down by mitochondrial fission and caspases [1]. If neuroprotection is prolonged by reducing mitochondrial fission or the caspase Dronc then axon regeneration is reduced [1]. We showed in this paper that overexpression of Fos also prolongs neuroprotection and reduces axon regeneration. We have added more information about this earlier work to the introduction to provide context for the experiment described here. The model proposed at the end (Figure 7G) I think can show how Fos is both needed for early injury signaling, but excess Fos can inhibit later axon regeneration.

2) The authors suggest that specially, FOS might be acting in early neuroprotection phase. I did not find any neuroprotection assay in this manuscript. Will there be higher frequency of degeneration for the injured neuron in the absence of fos? and opposing effect due to overexpression of FOS?

Fos’ role in neuroprotection has been established in our previous studies [1, 2]. In our neuroprotection assay, the cell was first subjected to axon injury and then a dendrite injury 8 hours later. In the control neurons, approximately 50% of the dendrites remained intact 18 hours after dendrite injury. However, expression of a dominant negative transgene of Fos reduced the number of intact dendrites significantly, demonstrating that Fos is required for neuroprotection. We have added more information about this earlier work to the introduction to try to make it clear that this is a backdrop for the current study.

3) In figure-2: It will be nice if authors describe the puc:GFP reporter assay. How it is an established reporter for injury response?
Is the phenotype of loss of puc is like the loss of fos or dlk?

We have added additional information about puc and use of puc-GFP as a reporter for axon injury signaling in the Results section that describes Figure 2. But yes, it is a validated reporter of early axon injury signaling.

We have not performed any loss of function studies for puc. However, based on the role of puc as a feedback inhibitor of JNK signaling [3] we hypothesize that loss of puc would resemble the phenotype for overexpressed Fos. We do not include this experiment here as we are focused on downstream transcription factors.

4) In Figure-4, What would be the effect of DLK knockdown on Fos homodimerization?

To perform this experiment, we would need to generate a Drosophila line with transgenes to monitor Fos-Fos interactions as well as drive RNAi expression. While we tried to generate this line, unfortunately we were unable to make a stable stock. Instead of this experiment, we tested whether Fos nuclear entry is dependent on DLK. We show this new data in Figure S2C and D. As Fos dimerization follows the same timecourse as nuclear entry, we expect that it would also be dependent on DLK.

5) In this report, the authors use the repolarization as an assay (injury done adjacent to cell body) for axon regrowth. what happens if the cut (axotomy) is done in a distal position in the axon? Whether axon regrows if the injury is done in the distal portion?

In that paradigm whether the knockdown of fos and jun will perturb axon regrowth to similar extent?

We agree that these are interesting questions and have investigated them in previous publications. When an axon is cut distally, the remaining axon stump is capable of regrowth [4, 5]. Moreover, both proximal and distal axon cut trigger the same DLK/JNK pathway [6]. Since Fos and Jun function downstream of the DLK/JNK pathway, it is likely that their knock down would similarly perturb axon regrowth following distal axotomy. However, as explained in the opening section of the results it is easier to quantitate axon regrowth after proximal axon injury.

Reviewer #3: The current manuscript from the Rolls lab addresses genetic and temporal relationships for transcription factors in the axon injury response in Drosophila sensory neurons. The question is important because many transcription factors have been shown to be induced by axon injury, but their functional roles, and relationships to each other have not been clear. The authors chose to compare the functions and subcellular localization of a handful of these conserved in axon injury in the Drosophila sensory neuron ddaE. The authors find that knockdown of Fos, Jun, ATF3, and STAT, all reduce axon regenerative capacity following axon injury. They then go on to show both differential regulation of these proteins by the DLK stress sensor as well as different regulatory relationships with each other. The questions addressed here are important and the authors made some new reagents: Fos-mNG, Jun-mNG and BiFC Fos that will be useful to the field. The major finding that Fos and Jun likely have different roles in the injury response is reasonably well supported. This paper would be clearer if it were tightened up around the central claims regarding Jun and Fos activity in axon regeneration. The data separating their roles in the regulation of STAT and Atf3 is confusing and detracts from the major claim.

We have chosen to continue to include the STAT and Atf3 sections as the other reviewers did not suggest removing them and we feel they make the story more complete. We have tried to address the suggestions below that are aimed at strengthening the data.

Several major concerns undercut my enthusiasm for a potentially important study. (1) The use of single RNAis for functional analysis. In two cases (Fos and Jun), the RNAis were put in a heterozygous background for some (not all) assays, and the phenotypes were modified, emphasizing the fact that the RNAis give only partial knockdown. This really undercuts confidence in the results—especially when the goal of the experiences (to order a pathway of genes with quite similar phenotypes) very tricky.

We have added additional RNAi lines for all conditions where we previously had only one genetic background tested for axon regeneration as suggested by the reviewer (Figure 1). We also tested the efficacy of STAT and Atf3 RNAi lines by examining their effect on STAT-GFP and Atf3-GFP. The two RNAi lines that have a stronger effect on STAT-GFP also reduce axon regeneration (Figure S1), and both Atf3 RNAi lines reduce Atf3-GFP (Figure S1) and axon regeneration (Figure 1). We only make conclusions from negative results with RNAi lines after we show that they are effective in reducing axon regeneration. We also pair these results with other types of experiments that support later functions of STAT and Atf3; for example STAT-GFP only increases in the nucleus 24h after injury (Figure 6B) consistent with it not contributing to puc-GFP upregulation at 6h after injury.

(2) The authors seem to assume that Jun acts following injury, presumably based on phospho-Jun increases following injury in other systems. However, in this system, it doesn’t seem clear that Jun is required following injury. Is it possible it is required developmentally and establishes competence of the neuron to respond?

We show that Jun is required for axon regeneration (Figure 1C), that its dimerization state is dramatically altered by axon injury (Figure 4E and F), and that dimerization is regulated by DLK and JNK (Figure 5E). As it is both regulated by axon injury and required for regeneration I do not think that we have overstated any of our conclusions.

(3) The data that Jun controls Atf3 activation while Fos does not is not at all convincing.

We actually show that Jun and Fos have similar effects on Atf3 (Figure 7). We have also added new data showing that Fos and Jun overexpression both reduce nuclear Atf3 (Figure 7E and F).

(4) I am not convinced that Atf3 regulation makes sense in the pathway as presented since it is a transcription factor that presumably acts in the nucleus; yet, as proposed the pathway in this manuscript regulate its export.

We try to be very clear that while we can clearly show that nuclear export is controlled by injury and the other transcription factors we do not know how this relates to output: “Although we do not understand how this localization pattern maps on to Atf3 function, the injury-induced change allowed us to determine what players in the DLK signaling cascade it responds to.”

More detailed comments:
• They should use more than 1 RNAi line per gene. Or at a minimum, they need to provide validation of the single RNAi lines they used to ascertain function. They establish that Jun RNAi line they use does not strongly knockdown Jun levels because the regeneration phenotype is strongly enhanced in a Jun heterozygote. They similarly put the Fos RNAi into a het background for some analyses. But these background were not always used, and similar approaches were not taked for Atf3 and STAT.

We have added axon regeneration data using a second RNAi line for Atf3 and Stat (Fig. 1C) and have also validated these RNAi lines (Figure S1). These experiments require getting a lot of transgenes into the same animal, so it was not always possible to use the RNAi mutant combinations for Fos and Jun. The worry with partial knockdown is that you cannot draw strong conclusions from negative data. In most of the assays we actually get phenotypes with the RNAi lines even when not combined with a mutant so partial knockdown is not an issue here. Where it comes up is with the early injury puc-GFP response not requiring transcription factors other than Fos. However, this result is supported by subsequent analysis of dimerization and nuclear entry changes happening later than Fos.

• The authors propose that Fos is necessary for “early step of the axon injury response”, which they think is neuroprotection. This conclusion is based on the requirement for Fos in Puc induction and in the confusing ability of Fos OE to suppress regeneration. The data that Fos is required specifically for the early injury response versus DLK-dependent response is weak.

I think there may be some confusion here, so sorry for that. All steps of the injury response are DLK-dependent (see Figure 7G). Our claim that the early injury response is neuroprotection is based on several of our previous publications that focused on defining this response, determining how it was turned on and off and also delineating its relationship with axon regeneration [1, 7]. We have now tried to do a better job of introducing this foundation in the introduction. The ability of Fos OE to suppress regeneration was actually an important point in one of these earlier papers and we explored it more there [1]. Here the key new piece is that Jun does not have the same effect and so acts differently than Fos.

• Potentially useful new reagents to track subcellular localization of Fos and Jun (Fos-mNG and Jun-mNG). They use them to report on different time courses of Fos and Jun following injury. Before using them as reporters of Fos and Jun subcellular localization, they need to first evaluate whether the endogenous tags have impaired function of either Fos or Jun by testing whether axon regeneration is impaired in a homozygous tag background. Otherwise, it is not clear whether they are faithful reporters.

We have now checked to make sure that both Fos-mNG and Jun-mNG are fully functional. Since Fos and Jun are essential genes and have important roles in development, we tested whether we could generate homozygous viable lines in which the only copies of the gene are the ones with the mNG tag. Indeed, we could maintain animals with both transgenes in a homozygous state indicating they fully support function. We now include this information in the Results section.

• Jun is clearly required for axon regeneration, but its nuclear levels do not increase following injury. Is it possible that the temporal requirement for Jun is before injury such that Jun is required to establish competence for the neuron to regenerate its axon.

Jun has been reported to be constitutively nuclear in other systems and its nuclear levels are not regulated by dimerization or JNK signaling [8], so its nuclear localization is not expected to report on activation. What we do detect is a very striking reduction of homo-dimerization in response to axon injury indicating that Jun is regulated by axon injury and so likely has roles in the injury response.

• The comparison between the ability of wildtype and phosphomimetic Jun to reduce fluorescence from the BiFC-fragment-tagged Jun is uninterpretable unless the transgenes are inserted at the same genomic location. They should also test whether the reduction in BiFC signal is simply due to Gal4 dilution by testing the degree to which it is reduced via the introduction of another UAS transgene.- bump into supplementary!

Yes, we agree and have put this data into the supplementary information section.

• The idea that Fos regulates DLK signaling and Jun does not is clear. On the other hand, the authors should soften their claims that Fos and Jun regulate axon injury at different time points, as they are inferring this based on changes in nuclear localization. This is particularly problematic in the case of Jun where it is unclear what nuclear localization corresponds to functionally.

Again, I think there is some confusion about Fos/Jun and DLK signaling. Both are downstream of DLK: Fos nuclear entry requires DLK (Figure S2) and separation of Jun homodimers requires DLK (Figure 5A and B).

Nuclear localization is only one small piece of the data that separates Fos and Jun function, and the constitutive nuclear localization for Jun is similar to that reported in mammals. Here is some of the other data that supports different roles for Fos and Jun:

a. Jun is not required for puckered-GFP nuclear accumulation at 6 hpi, but Fos is.
b. Overexpressing Jun has no effect on axon regeneration, unlike Fos overexpression.
c. Jun changes dimerization state at 24 hpi but not 6 hpi, unlike Fos which changes its dimerization state at 6 hpi.
d. Fos overexpression reduces STAT-GFP nuclear entry in response to axon injury, while Jun overexpression does not.

• The model that STAT is controlled by Jun is not convincing. Its nuclear localization is only modestly affected by Jun, and this effect seems suppressed when the RNAi is put into a jun heterozygous background. These authors should comment on this. Additionally, there is not a meaningful difference in the nuclear STAT localization in jun knockdown in the het background versus fos knockdown. Thus, it is unclear why the authors assert that STAT is downstream of Jun and not Fos.

We see reduction of STAT nuclear levels after injury with three different Jun loss-of-function conditions so are quite confident in this result. The Jun RNAi line (BDSC 31595) used here, and all other assays in Figures 1C, 2B and 7D has no predicted off targets. The mutant background, however, is not as clean. The chromosome with the *Jun* null allele has three other mutations on it (we now specify this in the Figure legend). I think it is therefore best to look at what is consistent here: that all three Jun LOF backgrounds reduce nuclear entry of STAT after injury.

When doing this assay we found that in uninjured neurons reduction of Fos increases nuclear STAT. This is why we do not conclude much from the post-injury data in 6D and why we performed addition experiments to probe the effect of Fos on STAT. The data in Figure 6E-H all consistently indicates that Fos negatively regulates nuclear STAT. So we actually do think that STAT is downstream of both Fos and Jun, but that Fos negatively regulates it while Jun is required (Figure 6K).

• Inclusion of Atf3 in a unified pathway is confusing since Atf3 RNAi has the same effect on axon regeneration as the other genes studied in this ms, but DLK, Jun, Fos, and STAT all appear to negatively regulate its expression since Atf3 nuclear localization is increased when these genes are knocked down. As presented, these data do not appear consistent.

We agree that the nuclear exit and then reentry of Atf3 is not simple to interpret and try to be very clear about this (from the Discussion: “How the Atf3 nuclear exit then reentry that we observe after axon injury relates to function and overall levels is unclear at this point, but we made use of its injury-dependent nuclear exit as an assay to determine whether it responds to the other transcription factors.”). We can see that the difference between injured and uninjured cells is strongly reduced by knockdown of any of the other transcription factors (Figure 7C and D) supporting the model that the other transcription factors are required for Atf3 to respond to injury. While the data may appear inconsistent, we cannot change the behavior of Atf3 to fit a simpler model and think it is better to include it than exclude it.

References:

1.	Chen, L., et al., *Mitochondria and Caspases Tune Nmnat-Mediated Stabilization to Promote Axon Regeneration.* PLoS Genet, 2016. **12**(12): p. e1006503.

2.	Chen, L., et al., *Axon injury and stress trigger a microtubule-based neuroprotective pathway.* Proc Natl Acad Sci U S A, 2012. **109**(29): p. 11842-7.

3.	Bohmann, J.Z.a.D., *Thorax closure in Drosophila: involvement of Fos and the JNK pathway.* Development, 1999: p. 3947-56.

4.	Stone, M.C., et al., *Dendrite injury triggers DLK-independent regeneration.* Cell Rep, 2014. **6**(2): p. 247-53.

5.	Stone, M.C., et al., *Normal Spastin Gene Dosage Is Specifically Required for Axon Regeneration.* Cell Rep, 2012.

6.	Rao, K.S. and M.M. Rolls, *Two Drosophila model neurons can regenerate axons from the stump or from a converted dendrite, with feedback between the two sites.* Neural Development, 2017. **12**(1).

7.	Chen, L., et al., *Axon injury and stress trigger a microtubule-based neuroprotective pathway.* Proc Natl Acad Sci U S A, 2012. **109**(29): p. 11842-47.

8.	Schreck, I., et al., *c-Jun localizes to the nucleus independent of its phosphorylation by and interaction with JNK and vice versa promotes nuclear accumulation of JNK.* Biochem Biophys Res Commun, 2011. **407**(4): p. 735-40.
